# Concomitant Box Lesion Ablation for Atrial Fibrillation with a Standard Non-Irrigated Bipolar Radiofrequency Clamp: Simplified Approach Without Left Atriotomy

**DOI:** 10.21470/1678-9741-2023-0432

**Published:** 2025-02-11

**Authors:** Vasily I. Kaleda, Mikhail A. Snegirev, Vidadi U. Efendiev, Aleksander V. Piskun, Igor A. Batukov, Kamo E. Nazaryan, Oleg Y. Pidanov

**Affiliations:** 1 Department of Cardiac Surgery, Yudin Hospital, Moscow, Russia; 2 Department of Cardiac Surgery, Mariinsky Hospital, Saint-Petersburg, Russia; 3 Department of Cardiac Surgery, Davidovsky Hospital, Moscow, Russia

**Keywords:** Atrial Fibrillation, Surgical Ablation, Posterior Box Isolation

## Abstract

In this paper, the authors describe a simplified technique for concomitant left atrial posterior box isolation for atrial fibrillation using a standard non-irrigated bipolar radiofrequency clamp without opening the left atrium.

## INTRODUCTION

**Table:** 

Abbreviations, Acronyms & Symbols	
AF	= Atrial fibrillation
IVC	= Inferior vena cava
LAA	= Left atrial appendage
SVC	= Superior vena cava

Traditionally, concomitant left atrial posterior box isolation for atrial fibrillation (AF) requires left atriotomy. However, when the primary purpose of surgery does not require atriotomy (in such cases as aortic valve replacement or coronary artery bypass grafting), additional atrial approach required for ablation may be the reason why AF is undertreated in these patients^[1]^. Here we describe a less invasive approach for concomitant box lesion ablation with a standard non-irrigated bipolar radiofrequency clamp without left atriotomy.

## TECHNIQUE

**Fig. 1:**
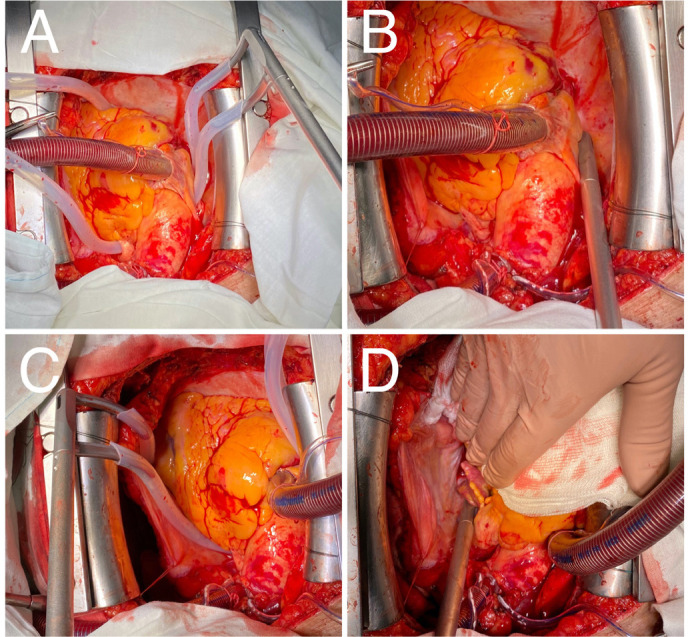
Intraoperative photos of the technique. A) and C) Silicon chest tubes are placed through the oblique and transverse sinuses of the pericardium posteriorly to the venae cavae to guide the ablation clamp; B) and D) rightand left-sided ablations are performed creating left atrial posterior box isolation.

**Fig. 2:**
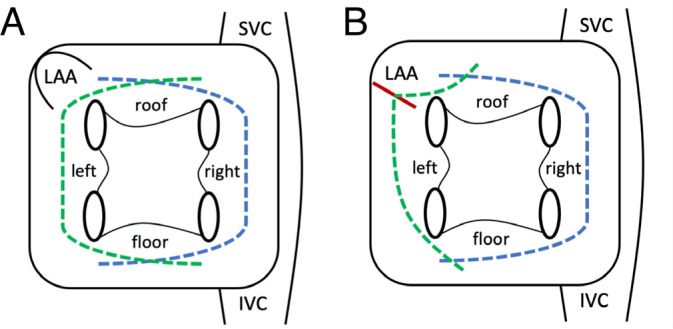
Schematic representation of the proposed techniques, view of the left atrium from behind. A) Completely closed technique. B) Left-sided ablation is performed through the base of LAA. Green dashed lines = left-sided ablation lines; blue dashed lines = right-sided ablations lines; red line = base of the LAA. IVC=inferior vena cava; LAA=left atrial appendage; SVC=superior vena cava.

The ablation is performed as a part of another cardiac surgery with the use of cardiopulmonary bypass. For success of the procedure, both venae cavae should be mobilized posteriorly to facilitate further clamp placing at the roof and the floor of the left atrium. Silicon chest tubes may be used to guide the Isolator Synergy ablation clamp (AtriCure, Mason, Ohio, United States of America) through the oblique and transverse sinuses of the pericardium (Figure 1A) and to allow its proper positioning at the atrium walls (however, silicon tubes are not really necessary for the procedure). The clamp is applied in the transverse fashion from the right to the left, posteriorly to venae cavae and anteriorly to the right pulmonary veins, creating the right side of the “box” (Figure 1B). On the next step, the clamp is applied in similar fashion from the left to the right, anteriorly to the left pulmonary veins, completing the box lesion (Figures 1C, 1D, and 2A).

We recommend multiple applications of the ablation clamp at each location to ensure transmural lesion. Care should be taken to cross the previous ablation lines which are clearly seen at the epicardium. When epicardial ablation lines are difficult to observe in case of severely enlarged heart, posterior box isolation may be confirmed with a high-frequency stimulation of the pulmonary veins. Finally, the left atrial appendage (LAA) is closed to reduce the risk of stroke, and primary purpose procedure is performed. When LAA is closed by cut and sew technique, left sided ablation may be performed through the base of the appendage (Figure 2B).

The full Cox-Maze lesion set may be then completed by adding right atrial ablation with a bipolar radiofrequency clamp and a cryoprobe through two purse-string sutures during the same procedure and by catheter mitral isthmus and coronary sinus ablation as a hybrid procedure.

## DISCUSSION

The described technique is relatively simple and adds little time to the procedure as it does not require opening the left atrium and extensive dissection around pulmonary veins.

However, this technique is not entirely new: epicardial left atrial posterior box isolation with a bipolar saline-irrigated radiofrequency clamp (Cardioblate Gemini-S, Medtronic, Minneapolis, Minnesota, United States of America) has been described as a part of thoracoscopic ablation for lone AF^[2,3]^. Recently, the same tool was used for concomitant AF ablation in patients undergoing off-pump coronary artery bypass grafting through a full sternotomy^[4]^.

More recently, a dedicated non-irrigated bipolar radiofrequency clamp (EnCompass, AtriCure, Mason, Ohio, United States of America) was designed for concomitant left atrial posterior box isolation without opening the left atrium^[5]^ and has already shown its efficacy against postoperative AF^[6]^. Compared to this novel device, our technique has similar simplicity and, probably, the same efficacy, but involves an older tool well known to cardiac surgeons. Moreover, the older tool allows for a lower economic burden per procedure, which is especially important in developing countries. However, for wider recognition of the technique, an analysis of the long-term efficacy is needed.

## CONCLUSION

The described technique makes concomitant AF ablation easier when the primary purpose of surgery does not require left atriotomy.
